# Optimized Gateway Placement for Interference Cancellation in Transmit-Only LPWA Networks

**DOI:** 10.3390/s18113884

**Published:** 2018-11-11

**Authors:** Hongxian Tian, Mary Ann Weitnauer, Gedeon Nyengele

**Affiliations:** 1 School of Electronic and Information Engineering Beijing Jiaotong University, Beijing 100044, China; 2 School of Electrical and Computer Engineering, Georgia Institute of Technology, Atlanta 30332-0250, GA, USA; mary.ann.weitnauer@ece.gatech.edu; 3 Department of Electrical Engineering Stanford University, Stanford 94305-2004, CA, USA; nyengele@standford.edu

**Keywords:** wireless sensor network, interference cancellation, capture, gateways location

## Abstract

We study the placement of gateways in a low-power wide-area sensor network, when the gateways perform interference cancellation and when the model of the residual error of interference cancellation is proportional to the power of the packet being canceled. For the case of two sensor nodes sending packets that collide, by which we mean overlap in time, we deduce a symmetric two-crescent region wherein a gateway can decode both collided packets. For a large network of many sensors and multiple gateways, we propose two greedy algorithms to optimize the locations of the gateways. Simulation results show that the gateway placements by our algorithms achieve lower average contention, which means higher packet delivery ratio in the same conditions, than when gateways are naively placed, for several area distributions of sensors.

## 1. Introduction

**Figure 1:**
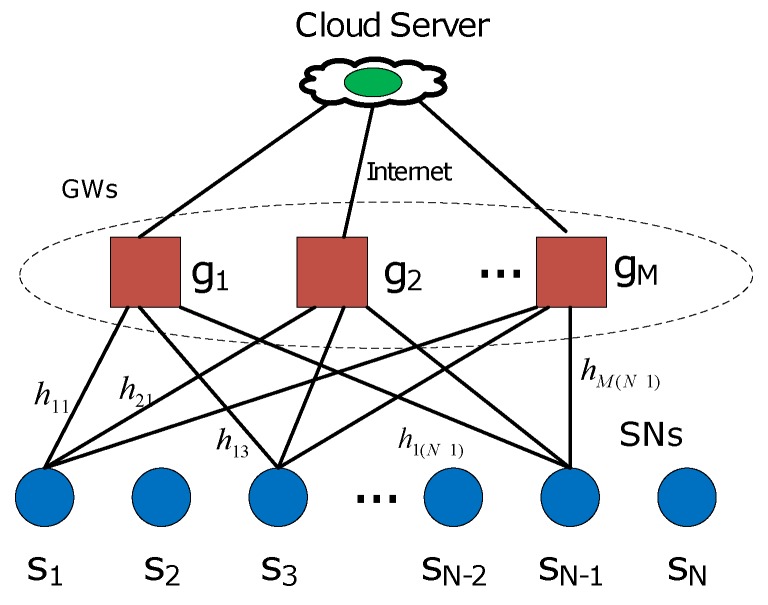
The network topology of the TO LPWAN.

The sensor nodes (SNs) in a low-power wide area network (LPWAN) are often required to be low cost and low energy, while the LPWAN should provide reliable communication and wide area coverage. LPWANs are an important technology for the Internet of Things (IoT) [1]. Several existing LPWAN solutions, such as the non-cellular systems of the SigFox [2], LoRa [3] and cellular narrow band IoT (NB-IoT) system of 3GPP [4], are being promoted. LPWAN application areas include smart cities, precision agriculture, wearables, transport, utilities and environmental monitoring [5]. In this paper, we study the sensor network, in which the sensors have one-hop to the gateways (GWs), which is known as star network topology (Figure 1), and in particular, the preferred placements of the GWs, when the GWs are capable of interference cancellation (IC).

Two of the main objectives in LPWAN design are to minimize power consumption on the sensors and minimize total system cost, including deployment, operation, maintenance, etc. Currently, most LPWANs are equipped with transceiver sensor radios. However, the receiver module of a transceiver often consumes more energy and is more costly than the transmitter [6,7,8]. Furthermore, many LPWAN applications contain hundreds or thousands of SNs, and have as their function to simply report sensed data to the server or cloud periodically and/or when an event is detected. Keeping the receiver turned off or removing it entirely not only saves the energy of receiver operation but also eliminates the energy overhead of medium access control (MAC) packets. Therefore, exploiting transmit only (TO) SNs in LPWAN can provide significant reduction in complexity, cost, and energy consumption [6,7,8].

The lack of receiver function in TO SNs has certain implications with regards to MAC. The TO SNs cannot perform carrier-sensing, as in the carrier sense multiple access (CSMA) systems, and data transmissions by these nodes are completely uncoordinated. This characteristic rules out the use of most existing MAC protocols such as IEEE 802.11 [9], B-Mac [10], S-Mac [11], and TSMP [12]. While ALOHA-based protocols are attractive for LPWANs because of their simplicity, lack of receiver rules out those protocols also, because acknowledgements cannot be received. Network time synchronization, which is the synchronization of the clocks on all the SNs in a network, is not possible for TO SNs, implying that time-division multiple access protocols cannot be supported. As in ALOHA-based protocols, collisions will be unavoidable in TO networks. Packet repetition provides reliability to TO SNs [13].

Advanced signal processing at the GWs allows most of the burden of TO channel conflict resolution to be shifted to the GWs, which simplifies the hardware and functionality of the SNs and drastically reduces the energy consumption in the SNs. To resolve the collisions, the GWs can perform capture and IC. Capture is the decoding of a packet despite interference. IC is the well-known process of subtracting the effects of a decoded packet from the stored soft samples of the received waveform, so that an underlying weaker packet may be decoded. Practical IC does not achieve perfect subtraction and the residual error power causes interference on the weaker packet. Many authors model the residual power from imperfect IC as a simple fraction of the power of the packet being canceled (e.g., [14]). This paper shows that this model of IC residual error implies quite particular preferred locations for the GWs.

The TO LPWA networks referred to in this paper, as illustrated in Figure 1, adopt a multi-gateway architecture, i.e., data packets are sent over wireless links from SNs to GWs, where the packets are decoded. Then, the GWs forward the packets to a server or the cloud via the Internet. We assume the GWs will coordinate data reception to reduce packet losses and maximize channel efficiency by providing diversity against fading and IC to mitigate packet collisions. Every SN is within a single hop of one or more GWs. Furthermore, we assume there are many more SNs than GWs, which makes packet collisions likely. For example, the topology of Figure 1 can represent quasi-stationary sensors used for monitoring and detection. The sensors may turn on receivers only to use GPS to determine their location, but turn off their receivers to save energy while they are stationary. If the device does not need to know its own location, the power consuming GPS chip can even be replaced by LPWAN localization algorithms based on signal strengths to base stations, such as fingerprinting and ranging. While stationary, the sensors can still make periodic reports of the monitored quantities or detected events. For example, the sensors could be on cars in a large dealership lot and report disturbances to the car. The locations of GWs are required to be strategically designed to maximize the chance of successful packet decoding, even if the packet suffers from a two-way collision. The direct motivation of this paper is coming from the Ph.D dissertation [8] of Firner, who aimed to maximize packet deliver ratio (PDR) of TO transmitters by optimizing the GW locations based on the capture effect in two-way collisions only. However, Firner [8] disregarded the IC function, i.e., only one packet can be captured and decoded when two packets are in collision following the algorithm of F-Embed in [8]. In contrast, with capture and IC, both collision packets can be decoded. This paper provides two algorithms to optimize GW locations for the TO LPWA network to minimize the average contention number, which is inversely proportional to PDR, when other factors are the same [8].

Network planning and optimization is a very active research area with a considerable amount of published work in many types of wireless communication systems. In [15], the optimal base station placement is obtained based on minimizing the sum of the ratio of the interference power to the signal power for all interesting points in the downlink for planning of a cellular network. To optimize average capacities of collaborated MIMO and distributed MIMO, the authors of  [16] employed the waveguide-multimode model and particle-swarm-optimization method. It obtained that distributed MIMO systems show desirable performance while significantly outperforming collaborated MIMO configurations. The authors of [17] proposed an algorithm specifically for LTE mixed-cell MIMO wireless systems, following a combinatorial approach and the optimization analysis based on the triplet of coverage, capacity and cost. In the research on the construction of WiMAX network, the authors of [18] transformed the relay station (RS) placement problem into a 0–1 binary programming problem in a two-hop IEEE802.16j network to maximize throughput. In the problem with huge input size, to find the sub-optimal solution, this paper proposes an efficient near-optimal placement solution for IEEE 802.16j WiMAX networks. Extending the work in [18,19] combining the condition of whether a subscriber station needs the relays, a model of three-hop IEEE802.16j network about station placement is proposed by limiting the placement location of RS.

In common wireless sensor networks (WSN), the designing and optimizing network structure is mainly focused on the cluster topology control and cluster head selection (e.g., [20,21,22]). Besides that, the authors of [23] focused on the topology control process for application nodes (ANs) and base stations (BSs), which constitutes the upper tier of two-tiered WSN. By proposing algorithmic approaches to place BSs optimally, the topological network lifetime of WSNs can be maximized deterministically, even when the initial energy provisioning for ANs is no longer always proportional to their average bit-stream rate. The obtained optimal BS locations are under different lifetime definitions according to the mission criticality of WSN. Aimed at providing the longest network lifetime, the authors of [24] proposed a modified integrated greedy method and a heuristic and greedy combinational method to find the base station location. In [25], location of BSs and distributed clustering by cluster heads are jointly optimized by the LEACH-C protocol to improve the energy consumption.

The rest of the paper is organized as follows. Section 2 introduces the system model and the main notation. In Section 3, we derive the optimized region of GW locations which meet the requirements on capture and IC for both pairs of SNs in conflict. In Section 4, two algorithms, the weight bipartite graph (WBG) algorithm and the pixels with gray levels (PGL) algorithm, are presented. The simulations of average contention for different GW placements are illustrated in Section 5 for different network topologies. Finally, Section 6 concludes the paper.

## 2. System Model

We consider a scenario with *N* SNs with the same transmit power and modulation scheme. The SNs are randomly located in a monitoring region with *M* deployed GWs that collect the data from the SNs.

In this paper, we model the channel with only path loss. Let the power received by GW *g* from the SN *m* be expressed as,

(1) $$\begin{array}{c} P_{r}\left(d_{m}\right)=P_{o}*{\left(\frac{d_{0}}{d_{m}}\right)}^{n}, \end{array}$$

where $d_{m}$ is the distance between the SN *m* and the GW *g*, *n* is the path loss exponent, and $P_{o}$ is the received power at a reference distance $d_{0}$ from the GW.

Packet collisions are unavoidable in a high density TO LPWAN. When *L* SNs transmit packets that collide at one GW, the overall signal at the GW will hence be the superposition of the *L* overlapping radio signals transmitted by the SNs, plus noise power $N_{0}$, with total power

(2) $$\begin{array}{c} \Lambda =\sum_{m=1}^{L}P_{r}\left(d_{m}\right)+N_{0}. \end{array}$$

### 2.1. Capture Effect

When the superimposed packets are received with significantly different powers, the so-called capture effect may take place, i.e., the strongest packet may be decoded by the GW despite a collision [26]. The signal-to-interference-plus-noise ratio (SINR) for the *j*th signal is defined as

(3) $$\begin{array}{c} \gamma_{j}=\frac{P_{r}\left(d_{j}\right)}{\sum_{m\neq j}P_{r}\left(d_{m}\right)+N_{0}}. \end{array}$$

We assume that a signal *j* may be “captured” by the GW and survive the collision if and only if $\gamma_{j}\geq \tau$, with τ>0 representing the so-called capture threshold of the system [26]. The capture threshold τ is a system parameter, whose value depends upon the sensitivity of the radio receiver. For instance, Firner e al. [27] showed a SINR of 6 dB was required for a Chipcon radio to capture packets, while Lee et al. [28] found that just 1 dB was enough for packet capture when the captured packet arrived before the interfering packet with an Atheros WiFi card.

### 2.2. Interference Cancellation

An effective approach to enhance the system capacity in the presence of signal interference is successive interference cancellation (SIC). Broadly speaking, SIC is an iterative reception scheme where signals are decoded one at a time, starting from the strongest, i.e., the one with the largest SINR. After the signal is decoded, its waveform is regenerated and subtracted from the aggregate received signal; then, the next strongest signal is decoded, regenerated, and subtracted; and so on [29].

Given error-free decoding, good regeneration of reconstruction requires accurate synchronization and a high quality estimation of the channel impulse response, which implies when these operations are imperfect, the signal cancellation leaves some residual power that increases the noise level experienced at the successive decoding stages. Furthermore, the finite precision of the analog to digital converter (ADC) at the receiver also reduces the effectiveness of each cancellation cycle. Following [30], we model all these idiosyncrasies of the interference cancellation process by assuming that the cancellation of a signal with received power *P* leaves a residual interference power of z∗P, where 0<z<1 is called the residual power factor. This model is clearly approximate, since the residual interference in practical SIC systems depends strongly on the SINR value of the canceled signal [26].

Considering that the decoded signal *j* is canceled from the overall received signal, leaving a fraction *z* of its power as residual interference, the SINR of next strongest signal *k* can be expressed as

(4) $$\begin{array}{c} \gamma_{k}=\frac{P_{r}\left(d_{k}\right)}{\sum_{h\neq j,k}^{L}P_{r}\left(d_{h}\right)+z*P_{r}\left(d_{j}\right)+N_{0}}. \end{array}$$

## 3. Single GW Placement for Two SNs

We first consider the simplest scenario, where only two SNs are deployed. When the packets transmitted by the two SNs collide, one GW in the optimum location should decode both of the packets successfully. In this section, the theoretical model where the GW should be optimally placed is deduced by IC after capturing.

### 3.1. Capture Circle

The contents of this section are summarized from [8], for the convenience of the reader and to establish notation. Considering two SNs located at $s_{1},s_{2}\in {ℜ}^{2}$ and one GW located at $g\in {ℜ}^{2}$, we use $s_{1},s_{2}$ and *g* as both their locations and identities for the sake of simplicity. A packet from $s_{1}$ will be captured and successfully decoded by the GW *g* if

(5) $$\begin{array}{c} \frac{P_{r}\left(d_{1}\right)}{P_{r}\left(d_{2}\right)+N_{0}}\geq \tau , \end{array}$$

where $d_{i}$ is the distance from GW *g* to SN $s_{i}$, and τ is the capture threshold, as discussed in the previous section. We omit noise term $N_{0}$ from the inequality in Equation (5) since it is expected to be negligible with respect to the other terms [26]. Then, we obtain that

$$\begin{array}{c} \frac{P_{r}\left(d_{1}\right)}{P_{r}\left(d_{2}\right)}\geq \tau \;⟺\;\frac{1/{\left(d_{1}\right)}^{n}}{1/{\left(d_{2}\right)}^{n}}\geq \tau , \end{array}$$

(6) $$\begin{array}{c} d_{1}\leq \beta d_{2}, \end{array}$$

where $\beta =\tau^{-1/n}$. With τ>1, then 0<β<1. We may write $d_{i}=∥g-s_{i}∥$, where ∥·∥ is the Euclidian norm of a vector in ${ℜ}^{2}$. Substituting into Equation (6) yields

(7) $$\begin{array}{c} ∥g-s_{1}∥\leq \beta ∥g-s_{2}∥. \end{array}$$

Squaring both sides of Equation (7) gives

$$\begin{array}{c} {∥g-s_{1}∥}^{2}\leq \beta^{2}{∥g-s_{2}∥}^{2} \end{array}$$

$$\begin{array}{c} ⟺\left(1-\beta^{2}\right)∥g^{2}∥-2g\cdot \left(s_{1}-\beta^{2}s_{2}\right)\leq -{∥s_{1}∥}^{2}+\beta^{2}{∥s_{2}∥}^{2}, \end{array}$$

where · means the inner product of two vectors. With 0<β<1, it follows that

(8) $$\begin{array}{c} ∥g-\frac{s_{1}-\beta^{2}s_{2}}{1-\beta^{2}}∥\leq \frac{\beta }{1-\beta^{2}}∥s_{1}-s_{2}∥. \end{array}$$

**Figure 2:**
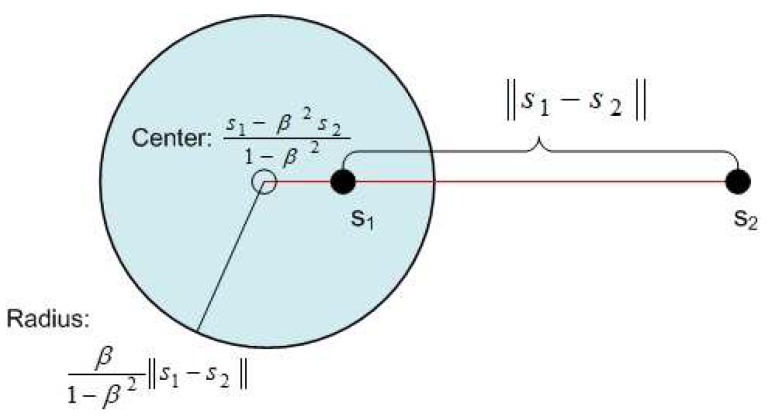
The capture region defined by center and radius, based on SN pair ($s_{1}$, $s_{2}$).

An interpretation of Equation (8) is that $s_{1}$ can be captured by *g* when $s_{1}$ and $s_{2}$ interfere, if *g* is inside a circle called the capture circle, with center $\frac{s_{1}-\beta^{2}s_{2}}{1-\beta^{2}}$ and radius $\frac{\beta }{1-\beta^{2}}∥s_{1}-s_{2}∥$ [8], as shown in Figure 2.

### 3.2. IC and the Decoding Circle

Suppose one packet in a colliding pair of packets is captured and canceled. Then, for the second packet to be decoded, the signal-to-interference ratio (SIR) of the second packet must satisfy

(9) $$\begin{array}{c} \frac{p_{r}\left(d_{2}\right)}{z*p_{r}\left(d_{1}\right)}\geq \tau , \end{array}$$

where $z*p_{r}\left(d_{1}\right)$ is the residual interference power after IC. Then, substituting Equation (1) yields

(10) $$\begin{array}{c} \frac{1/{\left(d_{2}\right)}^{n}}{1/{\left(d_{1}\right)}^{n}}\geq z*\tau ⟺d_{1}\geq {\left(z\tau \right)}^{\frac{1}{n}}d_{2}. \end{array}$$

Setting $\beta^{\prime }={\left(z\tau \right)}^{-\frac{1}{n}}$, we see that Equation (10) will convert to,

(11) $$\begin{array}{c} d_{2}\leq \beta^{\prime }d_{1}. \end{array}$$

Equation (11) shows the same expression as Equation (6) except that $d_{1}$ and $d_{2}$ have exchanged positions, and β is replaced by $\beta^{\prime }$. Then, following Equations (7) and (8), we have the following two cases.

**Figure 3:**
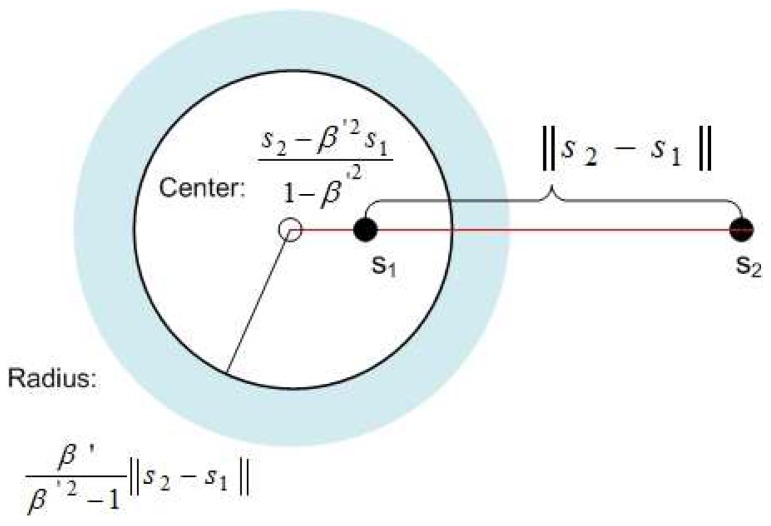
The blue regions outside of the circle are the ICD regions for $s_{2}$, following IC of $s_{1}$, for $\beta^{\prime }\geq 1.$

(1) if $z\leq \frac{1}{\tau }$, then $\beta^{\prime }\geq 1$,
  (12) $$\begin{array}{c} ∥g-\frac{s_{2}-{\beta^{\prime }}^{2}s_{1}}{1-{\beta^{\prime }}^{2}}∥\geq \frac{\beta^{\prime }}{{\beta^{\prime }}^{2}-1}∥s_{2}-s_{1}∥. \end{array}$$
  In this case, *g* will be *outside* a circle with center $\frac{s_{2}-\beta^{\prime 2}s_{1}}{1-\beta^{\prime 2}}$ and radius $\frac{\beta^{\prime }}{\beta^{\prime 2}-1}∥s_{1}-s_{2}∥$, as shown in Figure 3. This is the condition for $s_{2}$ to be decoded following IC of $s_{1}$, when $\beta^{\prime }\geq 1$. We refer to this circle as the IC and decoding (ICD) circle.
(2) if $z>\frac{1}{\tau }$, then $\beta^{\prime }<1$,
  (13) $$\begin{array}{c} ∥g-\frac{s_{2}-{\beta^{\prime }}^{2}s_{1}}{1-{\beta^{\prime }}^{2}}∥<\frac{\beta^{\prime }}{1-{\beta^{\prime }}^{2}}∥s_{2}-s_{1}∥. \end{array}$$
  In this case, *g* is inside a circle whose center is $\frac{s_{2}-\beta^{\prime 2}s_{1}}{1-\beta^{\prime 2}}$ and radius is $\frac{\beta^{\prime }}{1-\beta^{\prime 2}}∥s_{2}-s_{1}∥$. This area for *g* is illustrated as in Figure 2, with β replaced by $\beta^{\prime }$ and $s_{1}$, $s_{2}$ exchanged with each other, for $s_{2}$ to be decoded after IC of $s_{1}$.

### 3.3. IC and the Decoding Crescent

Considering interfering packets from SNs $s_{1}$ and $s_{2}$, assuming $s_{1}$ is the stronger packet, the optimized GW location should satisfy both the conditions for capture of $s_{1}$ and ICD of $s_{2}$. Therefore, combining Equations (6) and (11), we can get

$$\begin{array}{c} \frac{1}{\beta }\leq \frac{d_{2}}{d_{1}}\leq \beta^{\prime } \end{array}$$

(14) $$\begin{array}{c} ⟺\tau^{\frac{1}{n}}\leq \frac{d_{2}}{d_{1}}\leq {\left(z*\tau \right)}^{-\frac{1}{n}}, \end{array}$$

if and only if

(15) $$\begin{array}{c} \tau^{\frac{1}{n}}\leq {\left(z*\tau \right)}^{-\frac{1}{n}} \end{array},$$

i.e.,

(16) $$\begin{array}{c} z\leq \frac{1}{\tau^{2}}. \end{array}$$

The inequality in Equation (16) has some interesting practical implications. *z* and τ are controlled by separate physical mechanisms and separate parts of a packet. The minimum required SIR τ depends mainly on the choice of modulation and coding of the data. For example, a bandwidth efficient high-order quadratic amplitude modulation (QAM) with no error correction code can necessitate a high value for τ, whereas a power efficient high-order frequency shift keying modulation coupled with an error correction code can enable a low value for τ[31]. On the other hand, *z* is mainly controlled by the quality of the cancellation, which in turn, depends on the quality of synchronization and channel estimation, which is usually performed based on the packet ’s preamble and training sequence or pilot symbols [32,33]. For example, a long preamble and a long training sequence (and a low-Doppler channel, i.e., long coherence time) can enable a low value of *z*, whereas short versions of these will cause poor cancellation and a high value of *z*.

We next consider three cases involving *z* and τ≥1, especially: (1) $z\leq \frac{1}{\tau^{2}}$; (2) $\frac{1}{\tau^{2}}<z\leq \frac{1}{\tau }$; and (3) $z>\frac{1}{\tau }$. Three corresponding lemmas will prove that only the first case yields a non-empty set of GW locations, such that capture and ICD are possible. Define the ordered pair $\left(s_{1},s_{2}\right)$ to indicate that the packet from $s_{1}$ is to be captured, in the presence of interference from the packet from only $s_{2}$.

**Lemma** **1.** *For any ordered pair of SNs $\left(s_{1},s_{2}\right)$, if $z\leq \frac{1}{\tau^{2}}and\tau \geq 1$, the ICD circle will be inside the capture circle and there exists a region which satisfies both capture and IC requirements for GWs.*

**Proof.**  According to definition of capture circle and ICD circle separately in Equations (8) and (12), we can get the center and radius of them as follows.

Capture circle: center $C_{cc}=\frac{s_{1}-\beta^{2}s_{2}}{1-\beta^{2}}$ and radius $R_{cc}=\frac{\beta }{1-\beta^{2}}∥s_{1}-s_{2}∥$ and ICD circle: center $C_{ic}=\frac{s_{2}-\beta^{\prime 2}s_{1}}{1-\beta^{\prime 2}}$ and radius $R_{ic}=\frac{\beta^{\prime }}{\beta^{\prime 2}-1}∥s_{2}-s_{1}∥$ with $\beta =\tau^{-1/n}$ and $\beta^{\prime }={\left(z\tau \right)}^{-\frac{1}{n}}$.

The distance between the two circle centers is

(17) $$\begin{array}{c} D_{c_{1},c_{2}}=∥\frac{s_{1}-\tau^{-2/n}s_{2}}{1-\tau^{-2/n}}-\frac{s_{2}-{\left(z\tau \right)}^{-2/n}s_{1}}{1-{\left(z\tau \right)}^{-2/n}}∥. \end{array}$$

Set $\alpha =\tau^{2/n},\gamma =z^{2/n}$, then Equation (17) can be expressed

$$\begin{array}{c} D_{c_{1},c_{2}}=∥\frac{\alpha s_{1}-s_{2}}{\alpha -1}-\frac{s_{2}\alpha \gamma -s_{1}}{\alpha \gamma -1}∥. \end{array}$$

Then, after some algebra operations, we have

(18) $$\begin{array}{c} D_{c_{1},c_{2}}=∥\frac{\alpha^{2}\gamma -1}{\left(\alpha -1)(\alpha \gamma -1\right)}∥*∥s_{1}-s_{2}∥. \end{array}$$

On the other hand, the difference between the radii of the two circles is

(19) $$\begin{array}{c} D_{r_{1},r_{2}}=∥\left(\frac{\tau^{-1/n}}{1-\tau^{-2/n}}-\frac{z^{-1/n}\tau^{-1/n}}{z^{-2/n}\tau^{-2/n}-1}\right)∥*∥s_{1}-s_{2}∥. \end{array}$$

After substituting *z* and τ, and more algebra, we have

(20) $$\begin{array}{c} D_{r_{1},r_{2}}=∥\frac{\alpha^{1/2}\left(\alpha \gamma -1\right)+\alpha^{1/2}\gamma^{1/2}\left(\alpha -1\right)}{\left(\alpha -1)(\alpha \gamma -1\right)}∥*∥s_{1}-s_{2}∥. \end{array}$$

If we can prove that $D_{r_{1},r_{2}}\geq D_{c_{1},c_{2}}$ and $R_{cc}\geq R_{ic}$, we can prove that the ICD circle will be inside of the capture circle. Define

(21) $$\begin{array}{c} F\left(\alpha ,\gamma \right)=\frac{D_{r_{1},r_{2}}^{2}-D_{c_{1},c_{2}}^{2}}{∥s_{1}-s_{2}{∥}^{2}}. \end{array}$$

(22) $$\begin{array}{c} ⟺F\left(\alpha ,\gamma \right)=\frac{\left(\alpha -1\right)\left(1-\alpha \gamma \right){\left(1-\alpha \gamma^{1/2}\right)}^{2}}{{\left[\left(\alpha -1\right)\left(\alpha \gamma -1\right)\right]}^{2}}. \end{array}$$

For τ≥1, it is obvious that $\alpha =\tau^{2/n}\geq 1$. We know that $z\leq \frac{1}{\tau^{2}}$; then, (τz)≤1/τ≤1, and $\alpha \gamma ={\left(z\tau \right)}^{2/n}\leq 1$. Therefore, F(α,γ)≥0.

Similarly, set $\alpha =\tau^{2/n},\gamma =z^{2/n}$ and define

(23) $$\begin{array}{c} G\left(\alpha ,\gamma \right)=\frac{R_{cc}^{2}-R_{ci}^{2}}{∥s_{1}-s_{2}{∥}^{2}}. \end{array}$$

Then,

$$\begin{array}{c} G\left(\alpha ,\gamma \right)=∥\frac{\alpha^{1/2}}{\alpha -1}{∥}^{2}-{∥\frac{{\left(\alpha \gamma \right)}^{1/2}}{1-\alpha \gamma }∥}^{2}. \end{array}$$

(24) $$\begin{array}{c} ⟺G\left(\alpha ,\gamma \right)=\frac{\alpha \left(1-\gamma \right)\left(1-\alpha^{2}\gamma \right)}{{\left[\left(\alpha -1\right)\left(1-\alpha \gamma \right)\right]}^{2}}, \end{array}$$

where $\gamma =z^{2/n}$ and 0≤z≤1; then (1−γ)≥0. Because $z\leq \frac{1}{\tau^{2}}$ and $(1-\alpha^{2}\gamma )\geq 0$, then, G(α,γ)≥0. Therefore, it is proven that the ICD circle will be inside of the capture circle.

**Figure 4:**
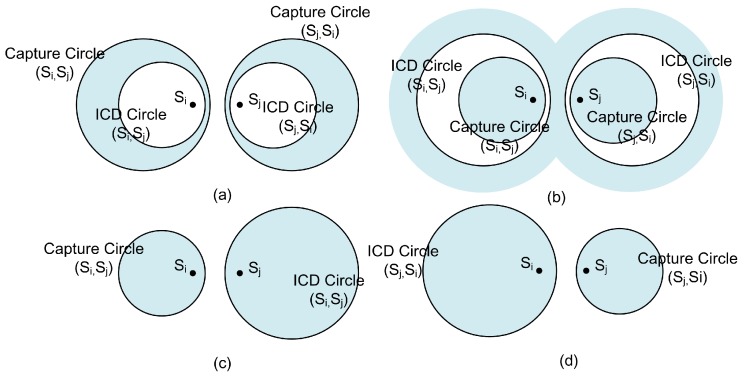
The capture and ICD Region for a pair of SNs $\left(s_{i},s_{j}\right)$, for τ>1 and: (**a**) $z\leq \frac{1}{\tau^{2}}$; (**b**) $\frac{1}{\tau^{2}}<z\leq \frac{1}{\tau }$; and (**c**,**d**) $z>\frac{1}{\tau }$.

Furthermore, $z\leq \frac{1}{\tau^{2}}\leq \frac{1}{\tau }$, for τ≥1, there exists a region which satisfies capture and IC requirements for GW according to Equations (8) and (12). Finally, Lemma 1 is proven as shown in Figure 4a. There are two symmetric shaded regions. A GW located in the shadowed region on the left will decode $s_{i}$ first and $s_{j}$ second. A GW located in the shadowed region on the right will decode $s_{j}$ first and $s_{i}$ second. We refer to each shaded crescent-shaped region in Figure 4a as a capture and ICD crescent or just a decoding crescents. □

**Lemma** **2.** *For any pair of SNs $\left(S_{i},S_{j}\right),\left(i\neq j\right)$, such that $\frac{1}{\tau^{2}}<z\leq \frac{1}{\tau }$, the capture circle will be inside of the ICD circle and there are no locations that satisfy both capture and ICD requirements for GW, given τ≥1.*

**Proof.**  According to Equation (22), for τ≥1, it is obvious that $\alpha =\tau^{2/n}\geq 1$. Furthermore, because $\frac{1}{\tau^{2}}<z\leq \frac{1}{\tau }$, then, (τz)≤1, and $\alpha \gamma ={\left(z\tau \right)}^{2/n}\leq 1$. Therefore, F(α,γ)≥0 and this means one circle will be inside of another circle.

Furthermore, from Equation (24), when $\gamma =z^{2/n}$ and 0≤z≤1, then (1−γ)≥0. Because $\frac{1}{\tau^{2}}<z\leq \frac{1}{\tau }$, then $(1-\alpha^{2}\gamma )\leq 0$ and G(α,γ)≤0. Now, it is proven that the capture circle will be inside of the ICD circle.

Finally, because $\frac{1}{\tau^{2}}<z\leq \frac{1}{\tau }$, for τ≥1, there is no region that satisfies both capture and ICD requirements for GW according to Equations (8) and (12), and Lemma 2 is proven, as shown in Figure 4b. The symmetric shaded region shows that capture region is inside of the capture circle and the ICD region is outside of the ICD circle. There are no overlap regions because the capture circle is inside of ICD circle. □

**Lemma** **3.** *For any pair of SNs $\left(S_{i},S_{j}\right),\left(i\neq j\right)$, such that if $z>\frac{1}{\tau }$, there will be no overlap of the capture circle and the ICD circle, given τ≥1.*

**Proof.**  Based on the same idea from Equation (22), for τ≥1, then $\alpha =\tau^{2/n}\geq 1$. Because $z>\frac{1}{\tau }$, then, (τz)>1, and $\alpha \gamma ={\left(z\tau \right)}^{2/n}>1$. Therefore, F(α,γ)<0 and there is no intersection between the capture circle and the ICD circle, as shown in Figure 4c,d for different capture order by $s_{i}$ and $s_{j}$.

Both packets transmitted by $s_{i}$ and $s_{j}$ in two-way collision will be decoded successfully by a GW inside of either one of the decoding crescents. Furthermore, it has to be noticed that, when $z=\frac{1}{\tau^{2}}$, the ICD circle and capture circle for $\left(s_{i},s_{j}\right)$ will be the same circle. □

### 3.4. Margins

In this section, we consider some SINR margins to improve the likelihood of correct decoding in the presence of variations in power levels, e.g., due to shadowing. In particular, these margins imply slightly larger or smaller circles; a capture margin and location margin will be suggested.

1. Capture Margin   

The capture margin is a small amount, ϵ added to the capture threshold τ, then, the SINR of a received packet in a GW, γ is shown in Equation (25).

(25) $$\begin{array}{c} \gamma =\frac{P_{r}\left(d_{m}\right)}{\sum_{i\neq m}P_{r}\left(d_{i}\right)+N_{0}}\geq \left(\tau +\epsilon \right). \end{array}$$

**Figure 5:**
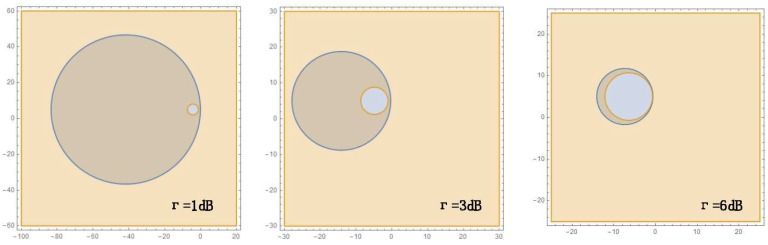
The capture margin of the τ with fixed *z*.

As shown in Figure 5, when *z* is held fixed while γ grows, that is, while the capture margin grows, it is clear that the radius of the capture circle will diminish. On the other hand, the radius of ICD circle will grow as the capture margin increases and both circles will become the same circle when $z=\frac{1}{\tau^{2}}$.

2. Location Margin   

**Figure 6:**
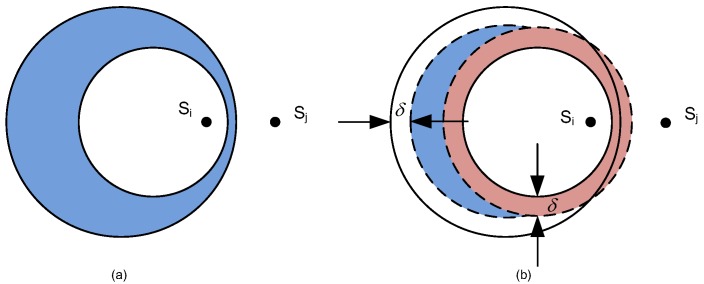
The location margin of δ. The blue region shows the location of GW: with location margin (**b**); and without location margin (**a**).

As shown in Figure 4a and Figure 6a, when the GW is inside the shaded region, i.e., it is inside of the capture circle and outside of the ICD circle, then any of the packets transmitted by $s_{i}$ and $s_{j}$ in collision could be decoded successfully by this GW. The shape of the shaded region is similar to a crescent. However, considering the uncertainties in power level, we should give some location margin between capture circle and ICD circle for optimized GW location, as shown in Figure 6b.

Suppose that we give the same value δ of location margin to decrease the radii of capture circle and increase the radii of ICD circle, then, if the minimum width of the ICD region is less than 2δ as in Figure 6a, we will get a real crescent region for optimized GW locations.

## 4. Algorithms for Multiple GWs Placement

We assume there are *N* SNs $\left\{s_{1},s_{2},\cdots ,s_{N}\right\}$ with known locations, and there are number of *M* GWs $\left\{g_{1},g_{2},\cdots ,g_{M}\right\}$ to be installed in this scenario to maximize PDR. We also assume $z\leq \frac{1}{\tau^{2}}$, as explained in Section 3.3, which implies that decoding crescents exist. Finally, without loss of generality, we set the location margin δ=0.

Our general approach is to superimpose the decoding crescents for all possible pairs of sensors, and place the GWs so that jointly, they serve the maximum number of sensor pairs. We propose two greedy algorithms that differ in the way they discretize the set of possible locations. The weight bipartite graph (WBG) approach uses intersection points of decoding crescents and the PGL approach limits potential GW locations to points or “pixels” in a rectangular grid.

### 4.1. Algorithm of Weight Bipartite Graph (WBG)

Inspired by [8], two sets of vertices are defined. The first set is composed of all possible optional points (OPs), where an OP is defined as point of intersection of the boundaries of a pair of decoding crescents. The second set of vertices contains all possible ordered pairs of SNs, such as $v\left(s_{i}s_{j}\right),\left(i\neq j\right)$. Since every pair of SNs generates two crescents, the ordering of pairs is necessary to distinguish between the two crescents. The first sensor in the ordered pair is the one that is captured. Weighted edges of the two subsets of vertices are applied according to the following rules.

1. If an OP is strictly inside of the capture region and outside of ICD region (inside of the ICD circle) decided by an ordered pair of SNs $\left(s_{i},s_{j}\right),\left(i\neq j\right)$, an edge with a weight of α exists between the vertices OP and $v(s_{i}s_{j})$. In other words, an edge is weighted by α if the OP is inside a capture circle but outside the decoding crescent.
2. If an OP is inside or on the boundary of the capture region and inside or on the boundary of ICD region (outside of the ICD circle) decided by an ordered pair of SNs $\left(S_{i},s_{j}\right),\left(i\neq j\right)$, which means in or touching a crescent, the edge between the OP and $v(s_{i},s_{j})$ has a weight of β.
3. Otherwise, there is no edge between the OP and $v(s_{i},s_{j})$.

**Figure 7:**
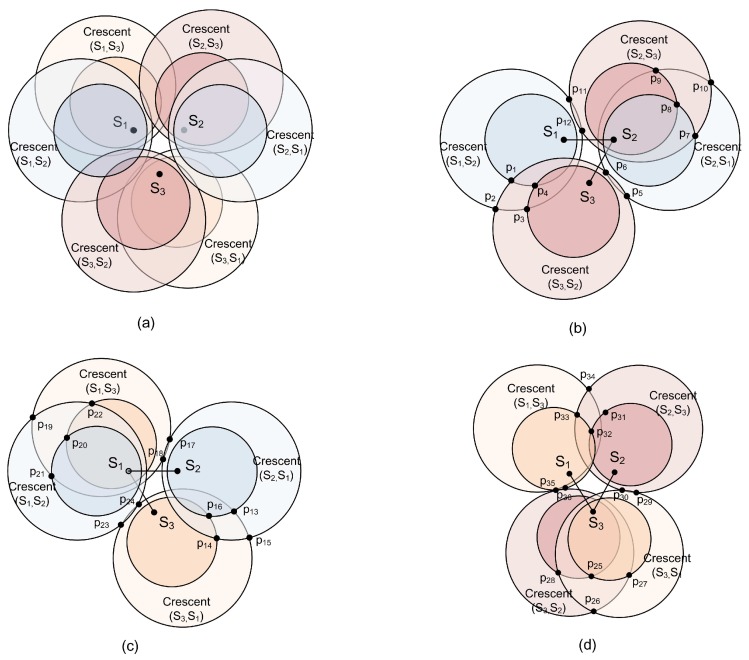
Crescent of each pair of SNs and location of 3 SNs and 2 GWs. The crescent region where the GW can decode both collided packets sent by the pair of SNs with capture effect and IC method, are shown in (**a**). In (**b**–**d**), the OPs are generated by the crescents corresponding to groups of sensor pairs $\{s_{1}$ and $s_{2}$, $s_{2}$ and $s_{3}\}$, $\{s_{1}$ and $s_{2}$, $s_{1}$ and $s_{3}\}$ and $\{s_{1}$ and $s_{3}$, $s_{2}$ and $s_{3}\}$, respectively, such as $\left\{p_{1},p_{2},\cdots ,p_{12}\right\}$ in (**b**).

We now walk through the WBG algorithm (Algorithm 1) for an example of three SNs and two GWs, when $z\leq \frac{1}{\tau^{2}}$, as illustrated in Figure 7. The capture circle and ICD circle for any ordered pair of SNs are calculated and we get the crescent region where the GW can decode both collided packets sent by the pair of SNs with capture effect and IC method, as shown in Figure 7a. In Figure 7b–d, the OPs are generated by the crescents corresponding to groups of sensor pairs $\{s_{1}$ and $s_{2}$, $s_{2}$ and $s_{3}\}$, $\{s_{1}$ and $s_{2}$, $s_{1}$ and $s_{3}\}$ and $\{s_{1}$ and $s_{3}$, $s_{2}$ and $s_{3}\}$, respectively, such as $\left\{p_{1},p_{2},\cdots ,p_{12}\right\}$ in Figure 7b. Because there are too many OPs in the example, some of them are omitted here. As an example of weight calculation, $P_{11}$ in Figure 7b is on the boundary of two decoding crescents, which gives two edges each with weight β. $P_{11}$ can also be located (it is not labeled) in Figure 7a, where it can be seen that $P_{11}$ is strictly inside the ICD circle of $\left(s_{1},s_{3}\right)$ and also inside the capture circle of $\left(s_{1},s_{3}\right)$. Therefore, $P_{11}$ gains an additional weight of α, for total weight of 2β+α.

**Algorithm 1** Algorithm of WBG

**Require:**
 *N* SNs locations, $\left\{s_{1},s_{2},\cdots ,s_{N}\right\}\in {ℜ}^{2}$;*M* the number of GWs;  
**Ensure:**
 The locations of number of *M* GWs, $\left\{g_{1},g_{2},\cdots ,g_{M}\right\}\in {ℜ}^{2}$.  1:Compute capture circle and ICD circle for each ordered pair of SNs $\left(s_{1},s_{2}\right),\left(s_{1},s_{3}\right),\cdots ,\left(s_{n},s_{n-2}\right),\left(s_{n},s_{n-1}\right)$ according to Equations (8) and (12) separately, and get the crescent region;  2:Compute the intersection points between any two ICD crescents decided by any two different pairs of SNs, and get the set $P=\{p_{1},p_{2},...,p_{i}\}$, which is the set of all of OPs  3:Construct a weight bipartite graph G = (P, S, E) conforming to the follows:5-1.$P=\{p_{1},p_{2},...,p_{i}\}$ is the set of vertices composed of OPs;5-2.$S=\{v_{\left(s_{1},s_{2}\right)},v_{\left(s_{1},s_{3}\right)},\cdots ,v_{\left(s_{1},s_{n}\right)},\cdots ,v_{\left(s_{n},s_{n-1}\right)}\}$ is another set of vertices decided by ordered SNs pairs;5-3.$E=\{e_{1},e_{2},...,e_{i}\}$ is the set of weighted edges connecting each $p_{i}$ and $v(s_{i},s_{j})$. If an OP, $p_{i}\in P$, is inside of the capture circle and inside of an ICD circle, set an edge with weight value of α for $e_{i}$; If an OP, $p_{i}\in P$ is inside of the capture region and outside of ICD circle, i.e., $p_{i}\in P$ is in the crescent, the edge $e_{i}$ is set by weight value of β; Otherwise, there is no edge between the $p_{i}$ and $v_{\left(s_{i},s_{j}\right)}$;4:**for**k=1 to *M* **do**5: Compute the sum of weight values of the edges that connect to each $p_{i}\in P$;  6: Get the OP, $p_{j}$, which has the maximum sum of weight values of edges;  7: Set the location of $k^{th}$ GW to be location of the $p_{j}$ with the maximum sum of weight values of edges;  8: For each of the $e_{j}$ connected to $p_{j}$, if the weight value of the $e_{j}$ is β, remove the edge $e_{j}$, vertices of $p_{j}$, and all of the connected vertices $v_{\left(s_{i},s_{j}\right)}$ and $v_{\left(s_{j},s_{i}\right)}$ even if they are not connected; if the weight value of edge $e_{j}$ is α, remove the edge $e_{j}$, vertices of $p_{j}$ and all of the connected vertices $v_{\left(s_{i},s_{j}\right)}$ only;  9: Get the new sets of *P*, *S* and *E*;  10:**end for**


**Figure 8:**
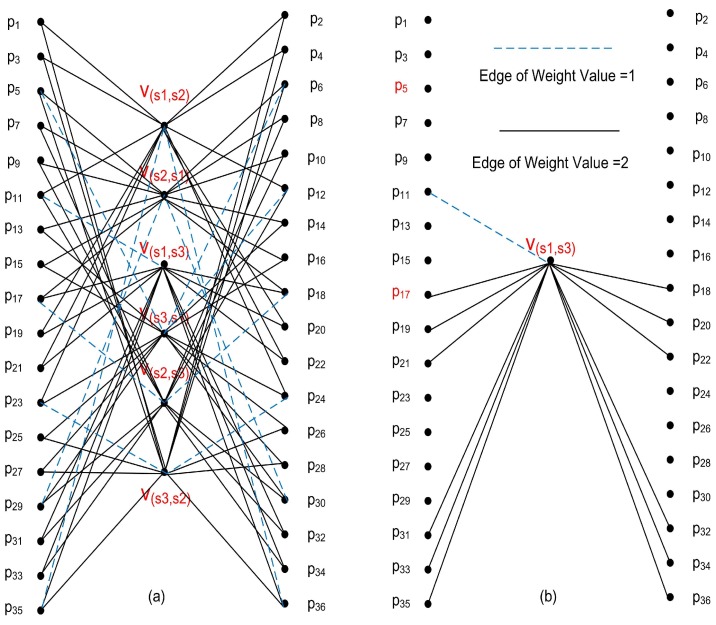
Example of the suggested WBG Algorithm with 3 SNs and GWs. (**a**) shows the complete bipartite graph, where α=1 and β=2. The weights of the edges joining $P_{11}$ and $V_{s_{1},s_{3}}$ and joining $P_{17}$ and $V_{s_{1},s_{3}}$ are 1 and 2, respectively, as shown in (**b**).

Figure 8a shows the complete bipartite graph, where α=1 and β=2. The weights of the edges joining $P_{11}$ and $V_{s_{1},s_{3}}$ and joining $P_{17}$ and $V_{s_{1},s_{3}}$ are 1 and 2, respectively, as shown in Figure 8b. The OP with maximum sum of weight values is selected as the first optimized location of GW, such as $P_{5}$ in Figure 8a, which has a sum of 5. In Step 8, assuming $P_{5}$ was selected, $V_{s_{2},s_{1}}$ and $V_{s_{3},s_{2}}$ are removed, because they are each connected to $P_{5}$ with an edge of weight 2. However, the reverse order vertices $V_{s_{1},s_{2}}$ and $V_{s_{2},s_{3}}$ must also be removed, because this first-placed GW will be able to decode all two-way collisions from $s_{1}$ and $s_{2}$ and all two-way collisions from $s_{2}$ and $s_{3}$, regardless of order. All edges connected to $V_{s_{2},s_{1}}$, $V_{s_{1},s_{2}}$$V_{s_{3},s_{2}}$ and $V_{s_{2},s_{3}}$ are removed, leaving what is shown in Figure 8b. Next, based on Figure 8b, the second optimized location of GW could be selected, such as $P_{17}$, because only one pair of SNs is left and $P_{17}$ connects to it.

### 4.2. Algorithm of PGL

With the increasing number of SNs, the WBG algorithm will become more and more time consuming, because the number of OPs grows rapidly with the number of SNs. Given of *n* SNs, there will be $O(n^{2})$ ordered SNs pairs with one capture circle and one ICD Circle for each pair. In the worst case, every crescent composed by a single capture circle and an ICD circle intersects with each other crescent except which decided by same pair of SNs with different order, then, there will be $O(n^{4})$ OPs. For every OP, we have to count how many capture circles and ICD circles it is in. Therefore, there are $O(n^{6})$ circles we count for $O(n^{4})$ OPs and $O(n^{2})$ capture circles and ICD circles.

**Figure 9:**
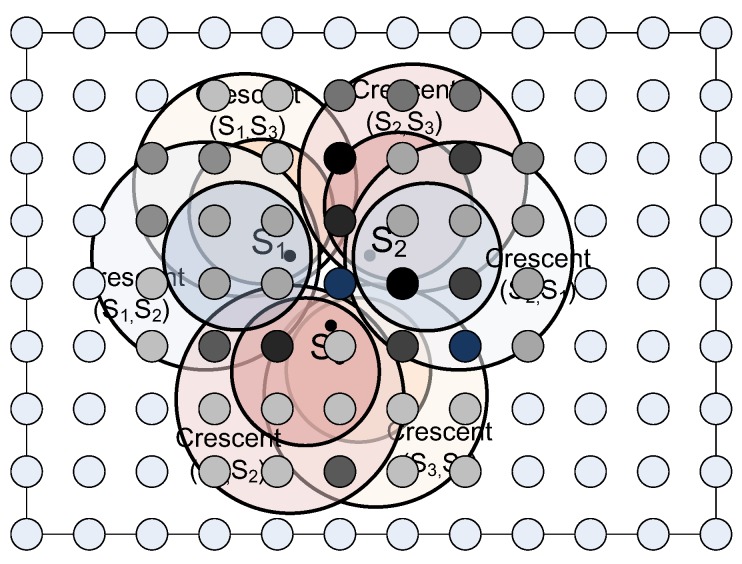
PGL Algorithm with 3 SN and 2 GW.

In the average case, every crescent does not necessarily intersect with every other, so the number of OPs will be less than that in the worst case. Even so, we still get a huge number of OPs to consider in the WBG algorithm. To decrease the number of OPs, which means to reduce the computational complexity, we may regard the whole potential area where the GWs could be deployed as a gray level image. Then, OPs are replaced by the pixels in the image and the edges with weight values are replaced by the gray level of each pixel, as shown in Figure 9. Therefore, the fast search algorithm of PGL is suggested as Algorithm 2.

It can be seen obviously in Figure 9 that different pixels located in different regions have different gray levels. The pixel with higher gray level means that the GW that location could decode more collided packets transmitted by two of the SNs in collision. Therefore, the single most optimized location of the GW will be at the pixel with the maximum gray level. After removing all of the SNs whose packets can be decoded in the collision, the algorithm begins a new computation cycle again, until the location of all of GWs are set optimally.

**Algorithm 2** Algorithm of PGL
**Require:**; *N* Number of SNs with known location; *R* Number of pixels in row;  *C* Number of pixels in column;  *M* Number of GWs;
**Ensure:**
 The locations of number of *M* GWs, $\left\{g_{1},g_{2},\cdots ,g_{M}\right\}\in {ℜ}^{2}$.
1:Compute capture circle and the ICD circle decided by each ordered pair of SNs $\left(s_{1},s_{2}\right),\left(s_{1},s_{3}\right),\cdots ,\left(s_{n},s_{n-2}\right),\left(s_{n},s_{n-1}\right)$;  2:Build the set of pixels, $P=\{p_{\left(i,j\right)}|0\leq i\leq R,0\leq j\leq C,\left(i,j\in Z\right)\}$ according to the number of pixels in row and column, and initiate all pixel gray levels to zero;  3:Build the set of ordered pair of SNs, $V=\{\left(s_{1},s_{2}\right),\left(s_{1},s_{3}\right),\cdots ,\left(s_{n},s_{n-2}\right),\left(s_{n},s_{n-1}\right)\}$ according to the number of the SNs;  4:**while**K≤M**do**5: **for** each pixel $p_{\left(i,j\right)}\in P$ **do**6:  **for** each capture circle and ICD circle **do**7:   If the pixel $p_{\left(i,j\right)}$ locates in the ICD circle, that is, outside of the decoding crescent but inside of the capture circle, add α to the gray level of this pixel and record the related pair of SNs $\left(s_{i},s_{j}\right)$ which decides the capture circle;  8:   If the pixel $p_{\left(i,j\right)}$ locates in the decoding crescent, add β to the gray level of this pixel, and record the related pair of SNs $\left(s_{i},s_{j}\right)$ which decides the ICD circle;  9:  **end for** 10:  change to next pixel $\left\{p_{\left(p,q\right)}\in P\right\}$;  11: **end for** 12: Sort all of the pixels {$p_{\left(i,j\right)}\in P$ } from the maximum gray levels to minimum;  13: Set the location of $K^{th}$ GW to be the location of the pixel $p_{\left(x,y\right)}\in P$ with the maximum gray levels;  14: Remove the pixel $p_{\left(x,y\right)}$ out of the set *P* and get the new set of the pixels $\left\{p_{\left(a,b\right)}\in P:p_{\left(a,b\right)}\neq p_{\left(x,y\right)}\right\}$;  15: Remove all of the recorded pairs of SNs $\left\{\left(s_{i},s_{j}\right)\in V\right\}$ connected to the pixel $p_{\left(x,y\right)}$ with gray level added by α in Step 7 out of the set, then renew the set *V*;  16: Remove all of the recorded pairs of SNs $\left(s_{i},s_{j}\right)$ and $\left(s_{j},s_{i}\right)$ in set *V*, connected to the pixel $p_{\left(x,y\right)}$ with either gray level added by β in step 8 out of the set, then renew the set *V* again;  17: K=K+1;  18:**end while**


The algorithm of WBG is expected to have better performance than PGL because of the precision of the OP locations, but at a cost of higher computational complexity. The PGL algorithm can significantly reduce the number of computations performed, although estimating the number of pixel points necessary for effective GW placement is not straightforward. However, the accuracy of GW locations will connect to the pixel density of the deployment area, as we show in Section 5.3.

## 5. Numerical and Simulation Results

In this section, to assess the performance of algorithms which we proposed, we firstly introduce an evaluation index, the “contention” of a SN. Then, we illustrate contention by comparing the contentions produced by a GW placed by the WBG and by two manual placements, for a simple scenario of three SNs and one GW. Next, we compare the performances of the WBG and PGL algorithms for different numbers of SNs and GWs.

### 5.1. Sensor Contention

The reason for applying TO to SNs for LPWAN is mainly to increase energy efficiency. However, the packet loss ratio (PLR) is impacted by many factors such as the duty cycle of each SN and the number of SNs in the network. Obviously, the PLR in a system with unchanged number of SNs will increase with the increased duty cycle. On the other hand, the PLR will decrease with fewer SNs for the same duty cycle. Following [8], we take SN contentions as the target metric to measure each SN and the whole TO system since it is not affected by traffic load, but only by the network topology.

In a TO network, the contention from the perspective of a SN is the number of other SNs which would prevent its packet from being decoded by any GW if the collision will happen. If there are *N* SNs in a network, and we say that SN A has a contention of *n*, it means that the packets transmitted by A can be decoded successfully (assuming two-packet collisions here), whether by capture directly or after IC, despite interference from any one of N−n sensors in N−1 sensors. In other words, SN A’s packet will be lost if interfered by any one of the other n sensors. For instance, assume there are 100 SNs (including SN A) in a TO network with one GW and no capture or IC. If A loses a packet when that packet collides with a packet transmitted by any one of the other 99 SNs, then, A’s contention is 99. As another example, if we build several GWs and each of the GW can perform capture and IC during a collision, and suppose A’s packet can be decoded if interfered with a packet from any one of 50 sensors, but not for the other 49 sensors, then A’s contention is cut down to 49, halved from the original setting. In our simulations, we compute the average contention, which is the average over the contentions of the SNs.

### 5.2. Simple Scenario

To test the abilities of the algorithms introduced above, we start from the simple scenario of three SNs on the corner of isosceles triangle and one GW. In this and the following sections, we assume the capture threshold, τ=3 dB, which is a system parameter, depended upon the sensitivity of the radio receiver. The path loss exponent n=3.2. In Section 2.2, we introduce the interference cancellation process by assuming that the cancellation of a signal with received power *P*, leaves a residual interference power of z∗P, where 0<z<1, is called the residual power factor, and z=0.1. In WBG algorithm, if an OP is inside of the capture circle and inside of ICD circle, set an edge with weight value of α; if an OP is inside of the capture region and outside of ICD circle, i.e., it is in the crescent, the edge is set by weight value of β. Otherwise, in PGL algorithm, if the pixel locates in the ICD circle, that is, outside of the decoding crescent but inside of the capture circle, add α to the gray level of this pixel; if the pixel locates in the decoding crescent, add β to the gray level of this pixel. In the two algorithms, we take α=1 and β=3. It is obvious that these values satisfy $z<\frac{1}{\tau^{2}}$, which means there exists intersection regions between the capture and ICD regions in WBG algorithm.

(1) Optimized GWs Placement 

**Figure 10:**
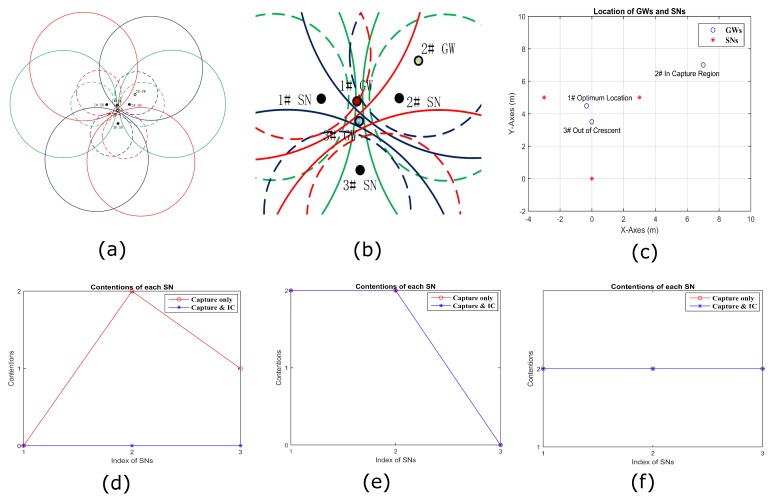
Contentions of each SN when a GW is placed at different locations. $1^{♯}$ GW is at the location by WBG placement, $2^{♯}$ GW is at the location of capture circle only and $3^{♯}$ GW is at location of out of any crescentm as shown in (**a**,**b**) by zooming in. (**c**) The coordinate location of SNs and GWs in different strategy. (**d**–**f**) The contention of each SN with three cases of the GW placement, respectively.

By algorithm of WBG, we can get an optimized placement of GW at the red-filled circle, labeled $1^{♯}$ GW in Figure 10b, which is a magnified view of the center of Figure 10a. Figure 10c shows the coordinates of all SNs and GW positions. The $1^{♯}$ GW is exactly in the region overlapped by the maximum number of crescents, where the average contentions for all of SNs is the least, which means it can decode the most packets in two-way collision. Figure 10d–f presents the contention of each SN by three cases of the GW placement, respectively. We can see in Figure 10d that contention of each SN is zero after capture and IC by the GW, which means that any packet transmitted by any of the three SNs could be decoded successfully in two-way collision. At the same time, it is obvious that the number of contention with capture only shown by the red line with circles in Figure 10d is more than that achieved by WBG algorithm.

(2) Placement GWs Manually 

To certify the results of the WBG algorithm, we place the GW location manually in the capture region but outside of the decoding crescent, as shown by $2^{♯}$ GW in Figure 10a,b. In Figure 10e, $1^{♯}$ SN’s contention is 2, which means the packet transmitted by it will be lost in any two-way collision. However, a packet transmitted by No. 3 could be decoded in any two-way collision with contention 0. Furthermore, it has to be noticed that the lines with capture only and capture with IC coincide because the GW is outside of all IC regions, but in one capture circle.

The $3^{♯}$ GW in Figure 10a,b, the blue-filled circle in the very center, is outside of all capture circles. Thus, the contention of each SN is 2, as shown in Figure 10f, i.e., none of packets transmitted by any SN can be decoded successfully in a two-way collision, whether by only capture or capture with IC.

### 5.3. Comparison of the WBG and PGL Algorithm

**Figure 11:**
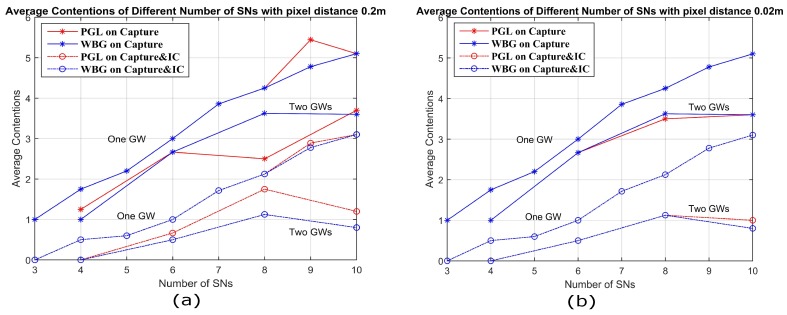
Average Contention for different number of SNs with one and two GWs, by WBG and PGL algorithms with capture only and capture with IC for different pixel densities.

In this section, we compare the two algorithms in terms of average contention for different pixel densities. Assume 3–10 SNs are placed at random in a 20×20 m${}^{2}$ network area. The average contention by two algorithms of WBG and PGL are shown in Figure 11. There are 100×100 pixels in Figure 11a and 1000×1000 pixels in Figure 11b. It is obvious that, as the pixel density is increased, the performance of the PGL algorithm approaches that of the WBG algorithm. In other words, the average contention of algorithms PGL and WBG, in the same network deployment space, will be more consistent, with more pixels at the same number of SNs and GWs. Therefore, the algorithm of PGL could have the enough accuracy for optimum location of GWs with enough pixels. We observe that PGL achieved a lower average contention for eight SNs with 100×100 pixels compared to 1000×1000 pixels. We attribute that aberration to the well-known fact that the greedy algorithm is not globally optimum and that the 100×100 locations may not have been a subset of the 1000×1000 locations.

### 5.4. Study of PGL for Larger and Different Network Topologies

In this and the following section, we compare the two algorithms for three network topologies, where we have specified a certain network size in terms of meters for the purpose of simulation. Because of the large number of SNs, we use only the PGL algorithm with 100×100 pixels for this study. Recall our assumption stated in the Introduction that each node in the network is within one hop of at least one GW and that there are many more SNs than GWs. Because of this, we have assumed that when one packet overlaps another packet, that the power of the weaker packet is so much greater than the noise that we can neglect the noise. Therefore, whether our topologies cover a large or small area, we still assume the weaker packet can be decoded on its own, because it is sufficiently stronger than the noise. In fact, the distances in the key equations, Equations (6) and (10), are in a ratio, so if the topology is scaled larger, the scaling factor cancels out and does not affect our results.

**Figure 12:**
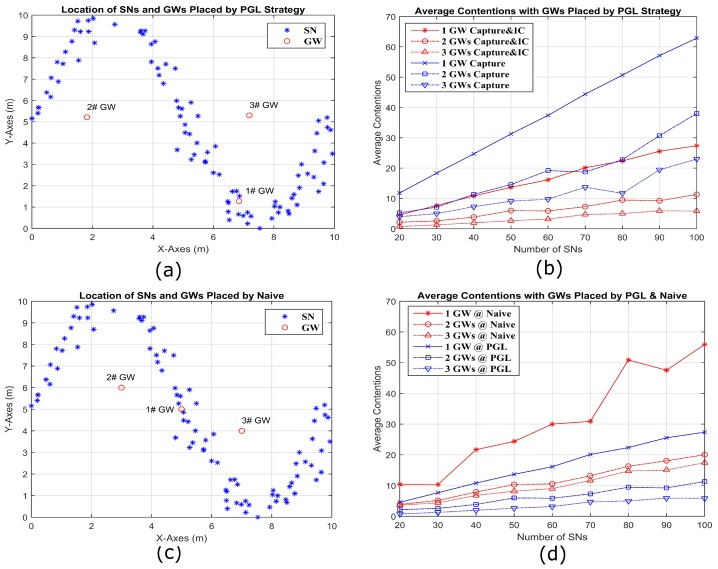
Simulation visualizations for three GWs placed by PGL (**a**) and by naive placement (**c**) along a sine wave composed by 100 SNs. (**b**) The average contentions comparing in different number of GWs by capture and IC to capture only separately; and (**d**) the average contentions for capture and IC comparing in different number of GWs placed by PGL and naive.

**Figure 13:**
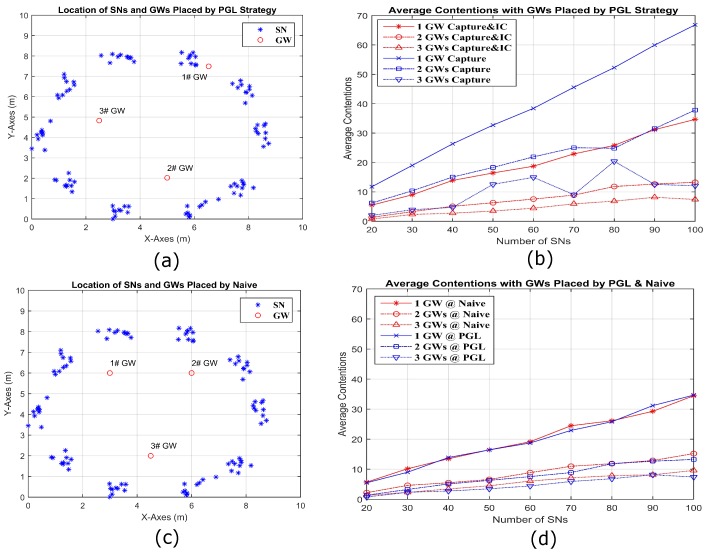
Simulation visualizations for three GWs placed by PGL (**a**) and by naive placement (**c**) in the circumference of a circle composed by 100 SNs. (**b**) The average contentions comparing in different number of GWs by capture and IC to capture only separately; and (**d**) the average contentions comparing for capture and IC in different number of GWs placed by PGL and naive.

**Figure 14:**
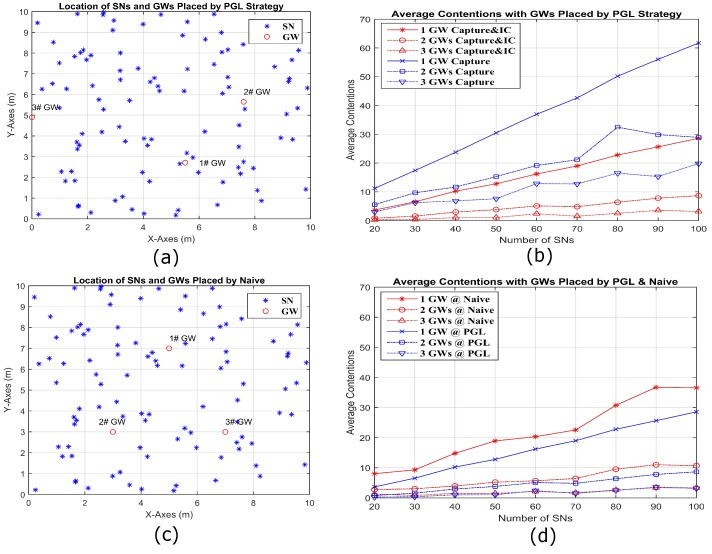
Simulation visualizations for three GWs placed by PGL (**a**) and by naive placement (**c**) in the circumference composed by 100 SNs in a uniformly random distribution. (**b**) The average contentions comparing in different number of GWs by capture and IC to capture only separately; and (**d**) the average contentions for capture and IC comparing in different number of GWs placed by PGL and naive.

We consider a sine-shaped topology in Figure 12, a circle-shaped topology in Figure 13, and a random topology in Figure 14, each in a 10 m× 10 m area. To make the circle topology, 100 SNs are grouped into 10 groups of 10 SNs each. The centers of the groups are equally spaced on a circle of radius 4 m. First, each SN is independently perturbed in angle from the center of its group by a zero mean Gaussian random variable (RV) of standard deviation of 4.5 degrees. From that perturbed location, each SN is again perturbed in both X and Y coordinates by iid zero mean Gaussian RVs with standard deviation 0.2 m. To make the sine topology, the initial X coordinates are distributed randomly over 9 m. The initial Y coordinates are the result of mapping the initial X coordinates through a sine curve that has an amplitude of 4.5 m and period of 9 m. These initial X and Y coordinates are then perturbed by iid zero mean Gaussian RVs with standard deviation of 0.27 m. Only one random outcome of each type of topology for a given number of SNs is used. Figure 12a shows the locations of 100 SNs deployed by sine shape and three GWs are placed by PGL algorithm. Figure 12b compares the average contention, for different numbers of GWs with capture and IC and with capture only. It is obvious that the average contention decreases significantly for capture and IC, compared to capture only. Figure 12c shows the deployed locations of 100 SNs in sine shape and three GWs by naive placement. In Figure 12d, it is clear that the average contention for 20–100 SN and 1–3 GW placed by PGL is always less than that based on naive GW placement. Figure 13 and Figure 14 show the similar conclusion with the SN deployed separately by circle and random topologies. Specifically, for the circle topology in Figure 13, we note that the naive and optimal GW placements are similar for two GWs, and the corresponding average contentions are close. For the random topology, we observe a larger difference between the naive and PGL placements.

### 5.5. Contentions versus GWs Number

**Figure 15:**
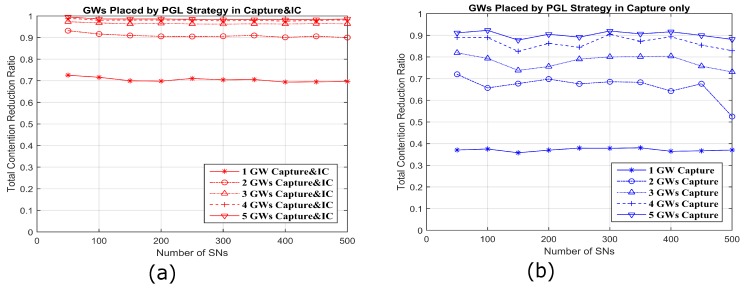
The results with GWs placed by the PGL algorithm with capture and IC and capture only both show that a given number of GWs can give a predictable contentions reduction even with more SNs. However, with capture and IC, the reduction of contentions has much more than that with capture only.

In this section, we consider the total contention reduction ratio by optimal placing of 1–5 GWs placed by PGL algorithm with capture and IC and capture only (Figure 15, which presents a comparison to Ref. [8]). The contention reduction ratio is defined $\left(N-\bar{n}-1\right)/\left(N-1\right)$, where *N* is the total number of SNs and $\bar{n}$ is the average contention. We note that N−1 is the contention of a SN when no capture is possible; in this case, any interference level is enough to prevent a packet from being decoded. The contention reduction ratio can be interpreted as the average fraction of SNs whose colliding packet will not cause a given SN’s packet to be lost. A perfect contention reduction ratio would be 1, which would mean that no SN’s packet would be lost in a two-way collision with any other SN’s packet. In this simulation, the number of SNs is increased up to 500 following a uniform random spatial distribution in a 100 m × 100 m area with the 1 m × 1 m pixel density. It is shown obviously in Figure 15a that a small number of GWs placed by optimal algorithm of PGL with capture and IC can decrease the contentions significantly. In other words, many deployed GWs is not necessary for some network layouts. For example, two GWs can reduce the contention level to above 90%, and 3–5 GWs provide little additional benefit on reducing the contention level. Therefore, the optimal number of GWs can be decided in the network infrastructure, based on this algorithm and other requirements.

### 5.6. Required GWs and Minimum Contentions

**Figure 16:**
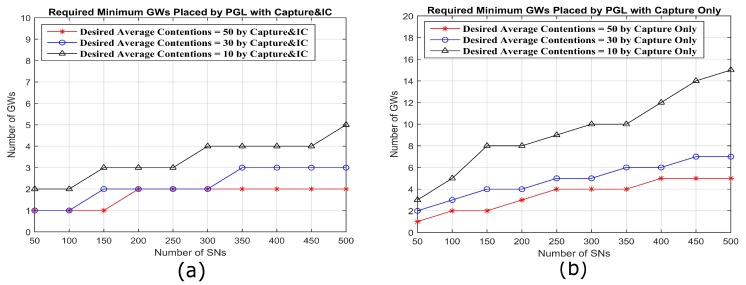
To maintain a desired minimum average contention, the number of GWs placed by the PGL algorithm, both with capture and IC and capture only, will grow with the increasing number of SNs. However, the required minimum number of GWs placed by PGL with capture and IC will be less than that with capture only.

Here, a desired contention level is specified for each given number of SNs. Then, the minimum required number of GWs to reach the desired contention level is determined, using the PGL algorithm, as shown in Figure 16a,b. Among the three desired average contention levels, 10, 30 and 50, the contention of 10 is a more strict requirement in 500 SNs than that of 50, which requires five GWs for 500 SNs compared to two GWs for the same number of SNs with capture and IC. On the other hand, with capture only, 15 GWs are required to get desired average contention levels of 10 with 500 SNs compared to five GWs at the same contention levels for capture and IC.

## 6. Conclusions

This paper provides a theoretical basis and a practical method to find the optimum location of GWs for transmit-only LPWA networks, assuming capture and interference cancellation. We follow the popular model that the residual interference from cancellation is a fraction of the power of the canceled packet. Based on this model and assuming a signal-to-interference or capture threshold for decoding, we derived the symmetric crescent shaped regions where a GW can be placed, to enable decoding of both packets in collision sent by two SNs. Based on this conclusion, to get the minimum average contentions, which means to achieve maximum PDR, we designed two greedy algorithms to find the optimized location of GWs. One algorithm is more precise but computationally complex. The other can be made to closely approximate the precise one, with much lower complexity. Based on simulation results, we showed that the lower complexity algorithm can get lower average contentions over different numbers of SNs, compared to the naive placement. Alternatively, the results show that the required number of GWs to perform the same average contention at a fixed number of SNs could be fewer with the optimal placement.

Our future work, besides optimizing the above WBG and PGL algorithms, will focus on the optimized location of GWs, adding the impacts of noise and multi-path fading. Furthermore, when more than two SNs are in collision, the approach in this paper cannot be applied. Then, a new algorithm for any number of nodes collision should be designed.

References
